# Supplementary material for: The effect of preferred music versus disliked music on pain thresholds in healthy volunteers. An observational study
Source: PLoS One. 2023 Jan 17;18(1):e0280036. doi: 10.1371/journal.pone.0280036 (PMC9844868; doi:10.1371/journal.pone.0280036)
Supplement: Attachment — Submitted filename: R2.0 Rebuttal letter.docx [file pone.0280036.s002.docx]

PONE-D-21-37630R1

The effect of preferred music versus disliked music on pain thresholds in healthy volunteers. A randomized observational study.

Dear Editor,

Thank you for the invitation to submit a revised version of our manuscript titled *“The effect of preferred music versus disliked music on pain thresholds in healthy volunteers. A randomized observational study”.*

We have included the following items:

- A rebuttal letter that responds to each point raised by the academic editor and reviewer(s).
- A marked-up copy of our manuscript that highlights changes made to the original version. However, we have left out the changes which were necessary due to the PLOS formatting guidelines. This because of readability.
- An unmarked version of our revised paper without tracked changes.
- We have uploaded our figure (Figure 1) to the PACE digital diagnostic tool to check and ensure we meet PLOS Requirements.
- In the manuscript it is specified where the minimal data set (including the syntax) underlying the results described in our manuscript can be found, with the DOI.

Below you will find our answers to the raised issues and questions.

Journal Requirements:

Please review your reference list to ensure that it is complete and correct. If you have cited papers that have been retracted, please include the rationale for doing so in the manuscript text, or remove these references and replace them with relevant current references. Any changes to the reference list should be mentioned in the rebuttal letter that accompanies your revised manuscript. If you need to cite a retracted article, indicate the article’s retracted status in the References list and also include a citation and full reference for the retraction notice.

*We have adapted the reference list to Vancouver style instead of the PLOS style in EndNote. Sorry for this mistake. Moreover, we have checked the reference list on completeness and correctness. No retracted papers were cited.*

**Reviewer comments:**

Reviewer #2: In relation to question 5 (writing style), there has been a significant improvement in the writing since the first submission. However, I noticed a few more errors/ambiguities that I have noted below.

*Dear reviewer, thanks for your comments and suggestions!*

**Introduction:**

Line 73-76 needs the citation.

*We added reference [11] to this line. (line 78-80)*

Line 83, the citation “[14]” would ideally need to be moved to after the author names, rather than at the end of the sentence to be consistent with how this was done on line 78 (“Bradt and colleagues [12]”). The alternative would be to adjust line 78 so that the citation is at the end of the sentence.

*We have replaced the references in the manuscript were necessary.*

Line 84 – do the authors mean that preferred music increased pain tolerance and lowered self-reported pain intensity?

*Our mistake, we have corrected it following your remark. (line 83-86)*

**Method:**

Line 188, NRS needs to be defined as it is first being used here.

*We have checked the acronym. (line 180)*

It might be good to include the ‘measurements’ section before the ‘Procedure’ section, as this defines all the acronyms and measures.

*We have placed the measurement section before the procedure section following your suggestion and checked the acronyms throughout the manuscript*

**Results:**

Line 266, use of the term ‘consummated’ is unusual here. This could be changed to ‘consumed’ or ‘ingested’.

*We have changed ‘consummated’ into ‘consumed’ (line 265)*

Lines 284-286, it’s usually good to also include some indication of dispersion e.g., SD or the range alongside the averages.

*We have added the standard deviations to the stated avarages. (line 282-286)*

Line 343, there is a spelling error/typo- “response”.

*We have corrected this typo.* (line 367-368)

**Discussion:**

Line 360, the word “also” can be deleted.

*We have deleted this word. (line 383)*

Lines 364-368, this sentence is quite wordy and makes an assumption that there is a ‘correct’ or ‘incorrect’ order of music choice. Although listening to disliked music first followed by liked music seemed to lead to better pain outcomes, describing the order of music in this way seems less objective.

*We have corrected this from:*

*“The differences in the outcomes of this study for perceived pain during listening to preferred versus disliked music were small but this sequence effect "comes along" for the correct order (listening to disliked music first and secondly to preferred music) and "goes off" for the wrong order (listening to preferred music first and secondly to preferred music).”*

*Into:*

*“The differences in the outcomes of this study for perceived pain during listening to preferred versus disliked music were small but this sequence effect "comes along" for listening to disliked music first and secondly to preferred music and "goes off" for listening to preferred music first and secondly to preferred music.” (Line 387-390)*

Lines 378-380, this sentence needs a citation.

*We added the required references. (Line 400-402)*

Line 399, it’s unclear what is meant by “no optimal characteristics for pain management were given” – it would be good to elaborate on this point more.

We agree this was unclear. We have re-written this as:

*“It is suggested that musical characteristics such as tempo, harmony, melody and volume might play a role. Based on a systematic review with meta-analyses [38] it could be concluded that instrumental music without lyrics is more effective in pain management of patients. However, based on this study it was not possible to identify the ideal music characteristics to be used in future pain management.****”*** *(Line 418-423)*

Line 429, there is repetition of the word “of”.

*We have corrected this in the manuscript. (Line 454)*
